# Exploring the impact of HIV and AIDS among marginalised farming communities in the Eastern Cape province, South Africa

**DOI:** 10.4102/hsag.v31i0.3294

**Published:** 2026-07-10

**Authors:** Ragosebo P. Sekopa, Robert T. Netangaheni, Lorraine N. Mntonintshi-Mketo

**Affiliations:** 1 Department of Health Sciences, College of Human Sciences, University of South Africa, Pretoria, South Africa; 2 Department of Sociology, College of Human Sciences, University of South Africa, Pretoria, South Africa

**Keywords:** exploring, impact, HIV and AIDS, farming, communities, marginalised

## Abstract

**Background:**

The HIV and AIDS epidemic on a global level continues to be a challenge in contemporary societies. The impact of HIV and AIDS on the workforce in the agricultural sector in Africa needs to be explored.

**Aim:**

The study aimed at exploring and describing the impact of HIV and AIDS on the farming labour force among marginalised farming communities at Oliver Reginald (OR) Tambo District in Eastern Cape province, South Africa.

**Method:**

The study utilised a qualitative research approach with exploratory and descriptive designs, employing in-depth interviews for data collection. Thematic analysis was conducted based on the framework established by Braun and Clarke.

**Results:**

The study found that HIV and AIDS have significantly negative impacts on labour, farming skills, income and food security in marginalised farming communities in the OR Tambo Municipality. Additionally, there are challenges in accessing healthcare services, leading to treatment defaults.

**Conclusion:**

HIV and AIDS directly affect the labour force, impacting households’ capacity to produce food or attend work to earn wages for food purchases.

**Contribution:**

The findings from the study would assist local stakeholders, farming forums and the Department of Rural Development and Agriculture in managing HIV and AIDS with respect to the farming labour force and farming during their programme revisions and implementation planning.

## Introduction

The farming community comprises farm owners and farmworkers in both crop farming and livestock farming. The farming community is not limited to families who practice non-commercial agriculture to sustain their livelihood (Roberts et al. 2025). The agricultural sector in African countries is predominantly situated in the rural areas, and most farmworkers are recruited from the local rural communities and neighbouring Southern African Development Countries (SADC). According to the World Health Organization (WHO), the African regions continue to be the most impacted, housing more than two-thirds of the world’s population living with HIV – that is, one in every 30 individuals (Joint United Nations Programme on HIV and AIDS [UNAIDS] 2023; WHO 2021). As of early 2025, approximately 31.6 million people globally are accessing antiretroviral therapy (ART). Despite this, the global AIDS response fell short of the 2025 target of 34 million, with roughly 10 million people (25%) out of the 40 million+ living with HIV not receiving treatment (UNAIDS 2025). HIV treatment access in 2024 was 77%, with 83% of females and 73% of males on ART.

Research cited in a Southern African Regional Poverty Network paper notes that in Kenya, an estimated 58% of all deaths of staff in the ministry of agriculture were due to AIDS, and 16% of Malawi’s staff were living with HIV and AIDS at the time of the study (Chakwizira 2024). The sub-Saharan Africa’s dependence on labour-intensive, rain-fed subsistence farming exacerbates its vulnerability to labour shortages. Research in Thailand indicates that HIV and AIDS has significantly affected agricultural productivity, with one-third of impacted rural families experiencing a 50% reduction in output (Mekouar 2021).

Based on recent UNAIDS reports, Eastern Africa and Southern Africa remains the epicentre of the HIV pandemic, accounting for over 50% of the global burden, despite significant progress in reducing new infections. South Africa, along with other high-burden countries like Mozambique and Tanzania, drives a large portion of these infections, with concentrated efforts needed to address the high incidence among young females (Mahy et al. 2021). These alarming spikes of new HIV cases are among farming communities in sub-Saharan Africa (Freer & Mudaly 2022).

Mlangeni et al. (2025) highlighted that there is also the presence of migrant workers from neighbouring countries such as Mozambique, Botswana, Swaziland and Zimbabwe who are working on the farms. People in the farming communities experience difficulties in accessing healthcare services based on the fact that the public healthcare facilities are located in distant places, and that makes it difficult to access on foot (Anowa et al. 2020). HIV and AIDS affect labour shortages and reduce productivity through increased worker illness and mortality, the diversion of caregivers from productive work, the loss of skills and experience and a general decrease in workforce quality and morale.

There is also a notable trend caused by the effects of HIV and AIDS, which sees productive time being lost to funeral attendance and mourning periods. It has been argued that SADC spend an average of 10% of their work time attending funerals instead of providing technical support to farmers (Chakwizira 2024). Daum et al. (2023) noted that the decrease in land use for cultivation due to labour shortages affects the agricultural mainstay of many communities. Agricultural skills and traditional farming methods are lost due to the mortalities of adults with agricultural knowledge, taking with them years of accumulated locally adapted knowledge (Kurebwa 2023).

The HIV and AIDS crisis significantly impacts farming communities in South Africa, influencing labour availability, food security and the transfer of farming skills across generations. A systemic approach that integrates health, agricultural and social support services is necessary to address these issues. Current research highlights a notable population and geographic gap in evidence for effective, tailored intervention programmes that overcome structural barriers to healthcare access for these communities (Mlangeni et al. 2023). Despite high HIV prevalence rates among farm populations, data on the efficacy of specific interventions remain insufficient (Mlangeni et al. 2023).

### Aim

The study aimed to explore and describe the impact of HIV and AIDS on the farming labour force and farming among marginalised farming communities in the Oliver Reginald (OR) Tambo District in the Eastern Cape province, South Africa.

## Research methods and design

The study employed a qualitative research approach with an exploratory and descriptive design. According to Bans-Akutey and Tiimub (2021), qualitative research is an approach to exploring while describing and understanding the meaning of an individual, group or social problem. A qualitative research approach using an exploratory and descriptive design is highly applicable to studying the impact of HIV and AIDS among marginalised farming communities in the Eastern Cape, South Africa, because it allows for an in-depth understanding of complex, subjective and context-dependent experiences that quantitative data cannot fully capture.

### Study setting of the farming communities

The study was conducted in the farming communities of the Eastern Cape in the OR Tambo District local municipalities (Port St. Johns, King Sabata Dalindyebo, Mhlontlo, Nyandeni and Ingquza Hill).

### Study population and sampling strategy

The study comprised 20 participants from the 5 farming communities in each local municipality of OR Tambo District Municipality. A non-probability sampling approach was utilised in this study. Convenience sampling was employed, whereby participants who were on duty and willing to participate were interviewed by the researchers. The researchers recruited and sampled the participants at five farming communities, each from OR Tambo District local municipalities (Port St. Johns, King Sabata Dalindyebo, Mhlontlo, Nyandeni and Ingquza Hill).

The study applied specific inclusion and exclusion criteria for participant selection. Inclusion criteria included individuals over 18 years old, both farmworkers and owners from the OR Tambo District Municipality, and those willing to participate without compensation. Exclusion criteria comprised individuals under 18 years, non-residents of the identified farms, and those unable to provide consent.

Before data collection, the researcher ensured participants understood the study’s purpose and were informed about risks, benefits, and their rights. Participants could freely agree or decline participation. Prior to semi-structured interviews, researchers secured completed informed consent forms and reiterated the study’s objective.

### Data collection methods and procedures

The researchers conducted a study using semi-structured interviews. The researchers created an interview guide in line with Saunders, Lewis and Thornhill (2019) to elicit detailed responses, starting each interview with a central question: *What is the impact of HIV and AIDS on the agricultural productivity, and food security of marginalised farming communities?*

#### Interview guide

(1) *In the past five years, what have you noticed as a change in the amount of food you produce or the size of land you cultivate caused by HIV and AIDS? If so, why?;* (2) *What farming knowledge or skills have you lost due to the passing of older family members?*, (3) *How have health-related expenses (medical, transport to clinics) impacted your household’s financial situation?*, (4) *Which assets did you have to sell (such as livestock, land and equipment) to cover costs related to illness or funerals?*, (5) *What kind of HIV and AIDS stigma and discrimination have you or others in the community experienced that affected your ability to work or access help?, and* (6) *What are the biggest challenges you face in accessing health services (e.g. ARV treatment)?*

### Data collection

An initial pilot interview was conducted prior to the main data collection to evaluate the effectiveness of the interview schedule in collecting the necessary data from participants. This pilot study included two research participants who conformed to the sample inclusion criteria.

The researchers utilised in-depth semi-structured interviews with a total of 20 participants to gather the data needed for their research. Data collection occurred from a total of 20 participants across different local municipalities in OR Tambo District. Interviews were conducted at the four local municipalities of OR Tambo District Municipality from 09 February 2022 to 01 March 2022. The research utilised structured individual interviews and qualitative observation for data collection, documenting verbal and nonverbal cues through field notes. Interviews, lasting under 60 min, were tape-recorded, and ethical standards were maintained with signed consent forms and clear explanations provided in participants’ native languages. Most participants spoke isiXhosa, with a couple using isiZulu, facilitating effective communication as the researcher was fluent in isiZulu.

### Data analysis

The procedure to analyse data was adapted from Braun and Clarke’s (2021) six steps of inductive thematic analysis. Step one focused on data familiarization through listening to audio recordings and reviewing notes. The audio, in isiXhosa, was transcribed and translated into English. Step two involved creating initial codes, followed by step three, which entailed searching for themes derived from interview data. In step four, themes were reviewed against research objectives. Step five defined the themes and sub-themes, culminating in a report in the final step. Consensus among researchers was achieved through structured collaboration methods such as researcher triangulation and peer debriefing.

### Trustworthiness

The researcher ensured transparency and credibility in the study through several key practices. Researchers planned in advance, disclosed conflicts of interest and obtained informed consent, committing to transparency by publishing results, sharing data and informing participants clearly. Prolonged engagement with participants facilitated trust and understanding, enhancing accurate data collection. Triangulation involved using multiple data sources and methods, such as interviews, surveys, observations and document analysis, to validate findings. Member checking confirmed findings’ accuracy with participants, while peer examination involved colleagues reviewing the research to identify biases, reinforcing the study’s credibility.

Dependability was established through an audit trail, documenting every research step for review consistency and replicability. Detailed documentation, the use of multiple analysts, reflexivity and external audits ensured trustworthiness. The study’s methods were described thoroughly for confirmability, allowing duplication by other researchers. Additionally, attention to field notes and non-verbal cues during interviews, along with describing the study environment, ensured transferability by outlining geographic and demographic boundaries.

### Ethical considerations

Ethical clearance to conduct the study was obtained from the University of South Africa’s College of Human Sciences with ethics clearance reference number: 90269543_CREC_CHS_2021. Researchers obtained permission from the Department of Rural Development and Agriculture, as well as from the owners of selected farms, to conduct their study. Before the study commenced, a comprehensive description outlining the goals and scope of the research was presented to the participants. Subsequently, informed consent was obtained from them, during which code names rather than real names were assigned to guarantee anonymity.

## Results

The demographic information of the 20 participants is presented in terms of age, gender, marital status, level of education, home language and main source of income (Table 1). Code names like participant 1, participant 2, etc., were used by the researchers to identify participants.

**TABLE 1:** Demographic information of the participants.

Participant number	Age (years)	Gender	Marital status	Level of education	Home language	Source of income
Participant 1	25	Female	Single	Grade 11	isiXhosa	Farmworker (receiving child grant for two children)
Participant 2	49	Male	Married	Below Grade 5	isiXhosa	Farmworker
Participant 3	61	Male	Widow	Below Grade 5	isiZulu	Project member (receiving old age grant)
Participant 4	52	Female	Widow	Below Grade 5	isiZulu	Project member (has three grandchildren with no birth certificates, daughter left home and unknown)
Participant 5	32	Male	Married	Grade 9	isiXhosa	Farmworker
Participant 6	25	Female	Single	Grade 10	isiZulu	Project member (receiving child grant for one child)
Participant 7	44	Male	Divorced	Grade 12	isiXhosa	Farmworker
Participant 8	30	Male	Married	Grade 11	isiXhosa	Project member
Participant 9	38	Female	Widow	Grade 9	isiXhosa	Project member (receiving child grant for four children)
Participant 10	27	Female	Single	Grade 12	isiZulu	Farmworker (receiving child grant for one child)
Participant 11	29	Female	Single	Grade 7	isiXhosa	Farmworker (receiving child grant for two children)
Participant 12	47	Male	Married	Grade 8	isiXhosa	Farmworker
Participant 13	61	Male	widow	Below Grade 5	isiXhosa	Farmworker (60 years and not receiving old age grant, waiting for identity document)
Participant 14	32	Female	Married	Grade 11	isiXhosa	Project member (receiving child grant for three children)
Participant 15	30	Female	Widow	Grade 12	isiXhosa	Project member (receiving child grant for two children)
Participant 16	49	Male	Divorced	Below Grade 5	isiXhosa	Farmworker
Participant 17	27	Female	Married	Grade 12	isiXhosa	Farmworker (receiving child grant for four children)
Participant 18	46	Male	Married	Grade 8	isiXhosa	Farmworker
Participant 19	47	Female	Widow	Below Grade 5	isiXhosa	Farmworker (has two children with no birth certificate and herself has no birth or ID)
Participant 20	61	Male	Widow	Below Grade 5	isiXhosa	Project member (60 years and not receiving old age grant)

ID, identity document.

### Key findings

Data were collected from a sample of 20 participants. Six main themes emerged from the data analysis.

#### Theme 1: Impact of HIV and AIDS on farming labour force and farming production

The participants were asked about the impact caused by HIV and AIDS on farming labour and farming production in the past 5 years, and what they have noticed as a change in the amount of food they produce or the size of land they cultivated.

Three participants reported low agricultural produce due to health issues, including HIV-related illnesses, tuberculosis lung infection and lack of rain, with one participant attributing the low yield not specifically to HIV illness.

The quotes presented below support the theme:

‘Due to the death of my two sons three years ago, I am unable to manage agricultural activities, and my wife suffers from diabetes and high blood pressure, preventing her from spending extended time in the garden.’ (Participant 2, 49, Male, Married)

‘I have tuberculosis in my lungs, but I do not have HIV. My blood was tested by doctors for viruses, but the results were negative. This tuberculosis is resistant; it simply won’t go away because I stopped taking my prescribed medication. Due to my inability to stay in the sun all day, I have postponed all of my farming efforts. As a result, last year’s harvest (2021) was extremely little, and my wife and kids are suffering and must rely on their neighbours for food when I run out. The social workers are making it difficult for me to register the three children under my care because my wife does not have identification.’ (Participant 12, 47, Male, Married)

‘Although there hasn’t been much rain, I can’t attribute the low produce to HIV infection. My wife and kids are HIV negative, but I am on ARVs and in excellent health. My four kids are all getting child grants.’ (Participant 18, 46, Male, Married)

#### Theme 2: Access to health services

The participants were asked about the biggest challenges they face in accessing health services, such as antiretroviral (ARV) treatment or other chronic medication.

Nine participants responded that being unable to access healthcare services is a threat of HIV and AIDS in the farming community.

The quotes presented below support the theme:

‘Lack of healthcare services care services is a challenging for people living with HIV with low resources.’ (Participant 11, 29, Female, Single)

‘HIV is a threat because of not having nearby healthcare services to collect ARVs and other chronic medication.’ (Participant 1, 25, Female, Single)

‘Healthcare services are hard to reach and many people cannot get tested for HIV, some fear because of not having adequate knowledge.’ (Participant 9, 38, Female, Widow)

‘Farm workers with HIV related illnesses are unable to collect ARVs because they do not have time to collect medication. Their bosses are refusing to give the day off or time offs.’ (Participant 15, 30, Female, Widow)

‘Being unable to access services is devastating. we are working long hours, when we are free on weekends the clinics are closed.’ (Participant 6, 25, Female, Single)

‘This can be all avoided if farm workers were provided with condoms weekly, and accompanied with HIV education.’ (Participant 7, 44, Male, Divorced)

‘Lack of awareness in the farming community ca result to farming being threatened. Farm workers are left out by the government system.’ (Participant 4, 52, Female, Widow)

‘Side effects of ARVs can be more severe on an empty stomach, causing some of us who HIV positive to stop taking them, for instance today I couldn’t take my medication because I don’t have food in my house, the only bread left was for my daughters. I cannot let my daughters to go to school on an empty stomach.’ (Participant 16, 49, Male, Divorced)

‘Not being educated leads to lack of important information like current development in chronic disease like HIV and corona virus [*COVID-19*], we rely on other people information.’ (Participant 13, 61, Male, Widow)

#### Theme 3: Stigma and discrimination

The participants were asked about the kinds of HIV and AIDS stigma and discrimination they are experiencing that are affecting their ability to work or access help.

Three participants expressed experiencing stigma and discrimination within their communities, particularly related to the fear of being seen at local clinics while obtaining treatment. This apprehension has led them to choose more distant clinics for collecting their HIV medications.

The quotes presented below support the theme:

‘Stigma often causes most of us to hide the illness rather than seek treatment, resulting in worsening health, inability to perform physically demanding agricultural labour, and, consequently, lower income.’ (Participant 17, 27, Female, Married)

‘Due to fears that confidentiality in the nearby clinic, I prefer to travel long distance to another clinic because no one knows me there. Even at work no one knows I am HIV positive.’ (Participant 14, 32, Female, Married)

‘A friend of mine tested positive for HIV and she shared it with a trusted elder community member, only to find out that this trusted person started to spread his status in the community, he was so devastated and he committed suicide.’ (Participant 19, 47, Female, Widow)

#### Theme 4: Financial burdens related to health and bereavement

The participants were asked which assets they had to sell (such as livestock, land and equipment) to cover costs related to illness or funerals.

Two participants disclosed that they sold their farming assets, including a truck and livestock, to support their families financially.

The quotes presented below support the theme:

‘I sold my truck because I was unable to afford its annual licence renewal and it was also experiencing mechanical issues.’ (Participant 3, 61, Male, Widow)

‘Two years ago, I sold five cows to fund my children’s university registration as they did not qualify for NSFAS. This year, two of my children were approved for funding, leaving one still without. Consequently, for the upcoming registration, I will need to sell at least two cows.’ (Participant 20, 61, Male, Widow)

#### Theme 5: Health-related expenses

The participants were asked about how the health-related expenses (such as medical and transport to clinics) impacted their household’s financial situation.

Three participants reflected on how these expenses have affected their overall financial stability and resource allocation.

The quotes presented below support the theme:

‘I am currently battling cancer and choose not to specify its location. I depend on various medications for my survival. My farm has been left untended due to my inability to afford labour, resulting in overgrown grass. I am considering selling the farm to support my family and cover medical expenses. I fear dying and leaving my wife and children without means to sustain themselves.’ (Participant 3, 61, Male, Widow)

‘I currently lack transportation to retrieve my medication. Although not prompted, I want to inform you that I am undergoing ARV treatment as well as treatment for high blood pressure. I have only seven pills remaining, which means the blood pressure medication can last for up to two weeks.’ (Participant 5, 32, Male, Married)

‘Due to financial constraints, I find it challenging to manage my expenses as the entirety of my old age grant is allocated towards basic needs such as food and electricity. Additionally, my wife will be eligible to apply for her old age grant in three years, which feels like a distant and difficult wait.’ (Participant 20, 61, Male, Widow)

#### Theme 6: Farming knowledge and skills

The participants were asked about farming knowledge or skills that they lost due to the passing of family members or colleagues at work.

Three participants reported challenges in locating qualified employees with the necessary skills essential for the successful execution of farming operations.

The quotes presented below support the theme:

‘In the context of a 100-hectare farm, I have faced significant challenges due to the COVID-19 pandemic. Initially employing 15 workers, the situation worsened with the loss of two employees who defaulted on their ARV treatment. The death of these employees severely impacted operations, leaving only nine workers. The remaining staff lack the complete skill set that the deceased employees possessed, which will likely affect farm productivity and management.’ (Participant 20, 61, Male, Widow)

‘I cannot claim to possess a skill; I regard myself merely as a farmer. The agricultural sector in South Africa will persist in its difficulties, as the farming communities are not provided with opportunities for skills development.’ (Participant 19, 47, Female, Widow)

‘It is challenging to find a skilled tractor driver, as the driver I previously employed had to take a long break from work. His absence has led to mechanical issues with the tractor, resulting in financial strain for my business. Consequently, I may need to consider reducing my workforce by laying off some employees.’ (Participant 3, 61, Male, Widow)

## Discussion

The study findings on the impact of HIV and AIDS among marginalised farming communities in the OR Tambo Municipality identified significant negative impacts on labour, farming skills, income and food security. Agricultural knowledge and innovation systems play an important role in maintaining natural resources management, and that maintenance is evident in the transition from conventional to agroecological system (Giagnocavo et al. 2022). The study conducted by Kountios, Chatzis and Papadavid (2024) revealed that agricultural knowledge and innovation systems have positive feedback in advancing the production sector towards sustainability. According to the European Commission (2019), knowledge and innovation play a key role in helping farmers and rural communities to meet substantial challenges, which include ensuring long-term food and nutrition security, bolstering environmental care and climate action and strengthening the socio-economic fabric of rural areas. Baiyegunhi (2024) acknowledged that there is a link between human capital (on-the-job training) and innovation behaviour of the farmers, which have positive impact on the productivity of the farm. The study found that human capital in the form of on-the-job training plays a significant role in knowledge and innovation creation and consequently farm productivity. Similarly, higher levels of human capital may lead to an increase in the number of innovative entrepreneurial farmers and products, resulting in productivity and economic development through the innovation channel (Diebolt & Hippe 2019). Results of the study further suggested HIV testing in the farming sector to manage the health of the farmworkers and farm owners. Therefore, investments in health management (like HIV testing and care) and education and training are both essential strategies for developing and maintaining the high-quality human capital needed to drive innovation, productivity and sustainable economic development in the agricultural sector. According to Mlangeni et al. (2023), HIV testing is important for early identification and treatment to increase health and reduce HIV-related illnesses. HIV testing among farmworkers and farm owners is very crucial; hence, many commercial farm employers already play an important but economic role in HIV and AIDS prevention and treatment through encouraging voluntary HIV testing and providing workers with information and transportation to clinics (Mlangeni et al. 2023).

Inability to access healthcare services within the farming community, which resulted in more people defaulting on taking their medication, was also revealed by the findings of the study as another direct impact of HIV and AIDS in the farming community. According to Harwell et al. (2022), farmworkers and farm owners are one of vulnerable working populations in precarious employment who are faced with economic deprivation, such as inequalities in accessing essential health services, including occupational health services, HIV prevention, treatment and care as compared to non-agricultural workers. Loganathan et al. (2019) issues of long distances to health facilities, conditions of employment, lack of health services and health system barriers contribute to the struggle of farmworkers and farm owners to access quality care. Empirical evidence suggests that farmworkers often exhibit a discrepancy between their knowledge of health risks and their actual safety practices, a phenomenon sometimes interpreted as ignoring or downplaying risks despite being aware of them (Durojaiye, Obisie-Nmehielle & Ibisomi 2021). Studies consistently show that a majority of farmworkers have adequate knowledge about HIV transmission and prevention methods like condoms (Durojaiye et al. 2021). However, this high level of awareness often does not translate into consistent safer sexual practices. For instance, one survey in South Africa found that 60% of all employees (and 53% of HIV positive employees) were not aware of their HIV status, and 25% of HIV positive employees who did know their status still did not use condoms (Durojaiye et al. 2021). It is therefore necessary for the farmworkers and farm owners to have a comprehensive programme designed to address this limited awareness specific to HIV and AIDS. The research has found gaps in knowledge within farming communities. One study in the Limpopo province of South Africa noted that some segments of the farming population do not receive the message from general awareness campaigns. Another study in the Tsitsikamma farming area found that a significant percentage of respondents did not know that HIV is a virus or what AIDS is, and a majority wrongly believed HIV could be transmitted through insect bites (Murwira et al. 2021).

### Strengths and limitations

The current qualitative research approach excels in gathering in-depth information about individuals’ experiences within specific cultural and socio-economic contexts, particularly regarding the impact of HIV and AIDS on farming communities. It effectively reveals the complex social, structural and individual factors, such as poverty, stigma, gender norms and access to healthcare. Qualitative methods are particularly useful for exploring sensitive issues like HIV-related stigma, where trust-building is essential. However, the study faced limitations, including restricted geographical coverage and potential selection bias resulting from the sampling technique.

## Conclusion

Illness and death affect prime-age, economically active adults, leading to a significant reduction in available farm labour. Crucial, often gender-specific, agricultural knowledge and skills are lost as adults die before passing them to the next generation. The farm employees needed some accessible voluntary counselling and testing (VCT) programme with regard to HIV and AIDS in order to prevent further new infections and manage the virus; hence, there is a test and treat programme. Therefore, the farmworkers and farm owners must be provided with a programme for HIV and AIDS testing, in addition to training. Policy on training for HIV and AIDS has to be followed by farm managers and supervisors so that this pandemic can be defeated. The farmworkers, farm owners and other employees must avoid discrimination against other farm employees who are either HIV positive or have AIDS.
